# #Lorrydeaths: Structural Topic Modeling of Twitter Users' Attitudes About the Deaths of 39 Vietnamese Migrants to the United Kingdom

**DOI:** 10.3389/fsoc.2022.787450

**Published:** 2022-08-11

**Authors:** Nga Than, Friederike Windel, Liza G. Steele

**Affiliations:** ^1^The Graduate Center, The City University of New York, New York, NY, United States; ^2^John Jay College of Criminal Justice, New York, NY, United States

**Keywords:** Lorry deaths, public opinion, immigration, social media, Britain, Brexit, topic modeling, attitudes

## Abstract

In this article, we analyze anti- and pro-immigrant attitudes expressed following the Essex Lorry Deaths tragedy in October 2019 in Britain, in which 39 Vietnamese immigrants died in a sealed lorry truck on their way to their destination. We apply Structural Topic Modeling, an automated text analysis method, to a Twitter dataset (*N* = 4,376), to understand public responses to the Lorry Deaths incident. We find that Twitter users' posts were organized into two themes regarding attitudes toward immigrants: (1) migration narratives, stereotypes, and victim identities, and (2) border control. Within each theme, both pro- and anti-immigration attitudes were expressed. Pro-immigration posts reflected counter-narratives that challenged the mainstream media's coverage of the incident and critiqued the militarization of borders and the criminalization of immigration. Anti-immigration posts ranged from reproducing stereotypes about Vietnamese immigrants to explicitly blaming the victims themselves or their families for the deaths. This study demonstrates the uses and limitations of using Twitter for public opinion research by offering a nuanced analysis of how pro-and anti-immigration attitudes are discussed in response to a tragic event. Our research also contributes to a growing literature on public opinion about an often-forgotten immigrant group in the UK, the Vietnamese.

## Introduction

Humanitarian tragedies tend to receive immediate attention and responses by the public on social media. Computational science research on Twitter responses to such events allows us to examine the public's responses in real time. Such research can access the immediate responses to social issues related to a tragic event, such as immigration, border control policies, white nationalist attacks, and more. While much of the research on public attitudes toward immigrants and border control involves analyzing survey data, the recent emergence of Twitter text data and appropriate methods to study social media data have provided new ways to investigate these topics. Social media platforms enable the public to express their attitudes about immigration issues, to make sense of tragic events, and to express collective trauma (Eriksson, 2016; Flores, 2017; Fischer-Pressler et al., 2019). In this paper, we focus on the Twitter responses to the Essex Lorry Death tragedy and analyze anti- and pro- immigration attitudes expressed within Tweets. With the passing of Brexit—a policy campaign that utilized anti-immigrant and pro-border control narratives—resulting in the departure of the UK from the EU in January 2020, the Essex tragedy coincided with a specific historic moment in the UK that was closely related to public discussions on immigration. The tragedy might have sparked a new round of public discussions about border control in the UK, immigration pathways, and the humanitarian consequences of harsh policies.

When the 39 Vietnamese immigrants died in the fall of 2019 in a lorry on their way to London, the Brexit debate was still ongoing and an extension of the transition period to Brexit was being discussed. A few months later, in January 2020, the UK formally left the European Union. The long debate about the Brexit referendum starting in 2016 highlighted the British public's disparate feelings about UK membership in the EU. The “remainers” argued that Brexit would have a detrimental effect on the UK economy: a decrease in investment, employment, and growth. In contrast, the “leavers” focused on maintaining the UK's sovereignty, ostensibly compromised by the EU's single market; they argued that the single market limited the power of the UK to choose who enters the UK labor market. The decision to leave the EU is frequently attributed to the UK's anti-immigrant attitudes (Viskanic, 2017; Zoega and Arnorsson, 2018).

In this context, this paper asks how and what kind of pro- and anti- immigration attitudes are expressed on Twitter. To explore this question, we examine English-speaking Twitter users' responses to the tragic Essex Lorry Death event. While survey research on public attitudes about immigrants in the UK has shown that the UK consistently ranks higher than other countries in their concerns about immigration and the spreading of anti-immigrant myths, we know little about how Twitter users' attitudes correspond to these concerns. We employ structural topic modeling, an unsupervised machine learning method that uncovers latent topics within a corpus of text documents, to analyze a dataset of 4,376 Twitter posts pertaining to attitudes toward immigrants in the wake of the tragic Lorry Deaths incident. This research shows how useful Twitter can be for public opinion research and the value of using computational social science to study social media. We also demonstrate a nuanced understanding of how pro-and anti-immigration attitudes are discussed in response to the tragic event. Finally, our study expands the limited literature on public attitudes toward Vietnamese immigration to the UK.

In the following section, we discuss the relevant literature regarding public opinion, and social media responses to tragic events. We then provide an overview of our data collection process, and the methods that we use in this paper. Next, we provide in-depth analyses of the themes that arose during the period after the tragic incident had occurred. Finally, we discuss the underlying meaning of each theme and its relevance to the broader literature, as well as the limitations of our approach.

## Literature Review

Social media research about humanitarian tragedies reveals that they provoke responses by social media users and that they can increase the frequency of tweets that spread anti-immigrant myths (Flores, 2017). Below, we discuss research on immigration attitudes in the UK and the events that led to Brexit to explore the context in which the Lorry Deaths incident occurred. Othering migrants and racialized others was central to the underlying dynamics of the Brexit referendum. We then examine literature on social media that elucidates the impact of humanitarian tragedies on public opinion toward immigration, specifically focusing on expressions of xenophobia and racism, as well as sympathy for migrants.

### Public Opinion on Immigration in the UK and the Brexit Context

The UK has consistently ranked higher than other European countries on anti-immigrant attitudes (Alfano et al., 2016; Blinder and Allen, 2016). Political opponents of immigration argue that immigrants disadvantage people born in the UK because they supposedly cause unemployment, depressed wages, or cuts to public services. Yet Alfano et al. (2016) show that immigrants, in fact, pay more in taxes than they receive in benefits, and help fund public services. Despite this evidence, anti-immigrant myths circulate widely in UK politics to justify policies such as Brexit; 52% of British citizens support increasing border controls to make immigration from the EU more difficult (Vasilopoulou, 2016). With the increasing racial and ethnic diversity in the UK, also called “superdiversity” (Vertovec, 2007), it is important to study specific immigrant groups. Since our paper is on the Essex tragedy in which 39 Vietnamese migrants died, it is important to look at the social science literature on Vietnamese immigrants to the UK. To our knowledge, there is scant research about Vietnamese immigrants to the UK, except for Barber's (2015) exploration of identities of the second generation of Vietnamese British.

Critical to the ultimate success of Brexit were the “long-standing racialized structures” underlying attitudes toward immigration and national belonging in the UK (Virdee and McGeever, 2018, p. 1804). In their study of discourses of the Brexit campaign, Virdee and McGeever (2018) show that the Leave campaign was driven by (1) a nostalgic feeling for empire and (2) a desire for “retreating from a globalizing world that is no longer recognizably ‘British’” (p. 1803). These discourses hinge on the existence of a category of “internal others” (p. 1803)—migrants, specifically Eastern European migrants, and racialized others, specifically Muslims, Black people, and Jewish people (Burnett, 2017)—against which the UK defines itself. After the 2016 EU membership referendum, racist violence in the UK rose, thus demonstrating the racist and anti-immigrant attitudes inherent to the Brexit campaign (Devine, 2018). In fact, the increase in hate crimes was greater than the increase observed after past terrorist attacks.

### Attitudes Toward Immigrants and Responses to Public Crises on Social Media

Determinants of anti-and pro-immigrant attitudes comprise a central topic in public opinion research. While extensive research has analyzed survey data on determinants of attitudes about immigrants, recent scholarship has investigated how the public express their attitudes toward immigration issues via social media, and whether social media can be used as a platform to measure public attitudes toward immigrants (Flores, 2017; Busch, 2018). Twitter is one of the social media platforms used to examine public opinion produced more spontaneously and in real-time than what surveys can provide (Flores, 2017; Rowe et al., 2021). Studying Tweets on this microblogging website provides researchers access to the ways that Twitter users make sense of events (Fischer-Pressler et al., 2019), share information (Bruns and Stieglitz, 2013), and create community, as well as the ways in which Twitter users (re)produce stereotypes of people and hateful content (Daniels, 2013; Flores Morales and Farago, 2021).

One example of research on anti- and pro-immigrant attitudes is Rowe's et al. (2021) analysis of migration-related Tweets during COVID-19 lockdowns in four European Countries and the US. It did not reveal increased anti-immigrant attitudes, and instead found high numbers of both pro- and anti-immigrant attitudes, thereby demonstrating increased polarization around migration. Flores Morales and Farago (2021) study on the (un)deservingness of undocumented immigrants shows that seemingly pro-immigrant Tweets further reinforce undocumented immigrants' exclusion via narratives of deservingness, such as those that portray “good” immigrants as essential workers and taxpayers, for example. On the basis of this research, our research asks what kinds of attitudes toward immigrants are expressed in the data set as Twitter users respond to the tragic event. This research also highlights the need for special attention to how the idea of “good” immigrants is constructed and how it implicitly reproduces exclusionary practices.

We analyze the pro- and anti-immigration attitudes expressed in response to the tragic Essex Lorry deaths incident. While public responses to tragic events and on immigrant attitudes have been studied, surprisingly scant research analyzes attitudes toward immigration in response to such humanitarian tragedies. Extant scholarship on the public's responses to tragic events on social media such as domestic terrorist attacks has examined social media's role in managing the public's collective trauma (Eriksson, 2016), how social media platforms such as Twitter helped in creating situation awareness related to the Stockholm shooting in 2011 (Steensen, 2018), and how the public collectively made sense of a sudden terrorist attack (Fischer-Pressler et al., 2019). Using sense making and terror management theory, Fischer-Pressler et al. (2019) were able to explain the German public's behaviors on Twitter after the Berlin terrorist attack.

Given this literature, we expect to find expressions of anti- and pro-immigrant attitudes in the data as they relate to the specific British Post-Brexit context. There is considerable evidence that policies like SB 1070 in Arizona, USA, or Brexit in the UK affect attitudes and behavior as they relate to immigration. As Flores (2017) showed, laws can encourage and mobilize groups that have already-existing attitudes, specifically anti-immigrant attitudes, to be expressed more publicly.

Whether it is the increase in expressions of opinion, a shift in attitudes, or anti-immigrant opinion veiled in deservingness language, scholars demonstrate that studying Twitter responses about public opinion on immigration and tragic events enhances our understanding of public attitude data and ways in which debates emerge in real-time regarding social issues like immigration and violent events. Furthermore, attention to discourses on anti- and pro-immigration can provide insight into how such attitudes are constructed. In extending this work, our research examines how Twitter users responded to the deaths of 39 undocumented Vietnamese immigrants in a lorry truck in Essex, UK, in 2019, and how they connect this tragic event to their attitudes toward immigration.

### Case Selection

On October 23, 2019, 39 undocumented Vietnamese immigrants were found dead in a lorry truck in Essex near London. Thirty-one were male and 8 were female and their ages ranged from 15 to 44, with an average of 27. Most of the victims were in their 20s, and 30s. The lorry had driven from Bulgaria to Belgium and then entered the UK from Belgium. The victims were first believed to be Chinese nationals because they carried forged Chinese passports. After intervention on social media from Vietnamese citizens who feared that the victims were their family members who had recently boarded a truck to the United Kingdom, the victims were identified as Vietnamese. One of the interventions was by a Vietnamese father who circulated a message from his daughter, which read: “I could not breathe. Sorry mom. My way to the foreign country has failed” (Parveen et al., 2019). The driver of the lorry truck, Mo Robinson, a Northern Irish man, was immediately charged with manslaughter. The incident shook the British public.

The victims mainly came from northern central Vietnam, which is considered the least developed part of the country, plagued by poverty, underdevelopment, and constant natural disasters. The victims had to pay a substantial amount of money to be trafficked into the UK, which left many families in debt. This event raised questions about human trafficking in the UK, as the public started to discuss the extent of the human trafficking industry that brings people into the UK in inhumane ways.

This event provides a case study through which we examine public discourses around border and immigration policy and how porous borders in the European Union shape immigration routes. By analyzing debates on social media, this paper provides data on how the British public responded to a tragic event that involved immigrants who without proper documentation crossed many levels of international borders and died during the passage.

## Data and Methods

### Twitter's Strengths and Weaknesses

This paper uses Twitter data to learn about the public responses to a tragic event that is deeply connected to questions of migration and human rights. We chose to use Twitter data because of its many unique advantages. Our main interest in this paper is about the pro- and anti- migration attitudes in the responses to this event. Twitter is a public social network site which, in theory, is accessible to all. While Twitter is widely used across countries, it is not representative of national populations. In the UK, for example, the total number of Twitter users was 19.05 million in October 2021 (Strugar, 2022). Twitter demographics for the UK show that users are more often male, predominantly young (yet still older than previously thought), and that class plays an important role in the use of Twitter (for example, professionals who occupy “managerial, administrative, and professional occupations” are over-represented) (Sloan, 2017, p. 9). Thus, Twitter users' characteristics are not representative of the general public. Nonetheless, Twitter is an important place to examine discourses on social events since the societal discourses that are present there may influence people's lives, politics, and other social spheres.

In addition to the discourses present on Twitter, communication scholar Papacharissi (2015) emphasizes the role of “affect” on Twitter as social media users become mobilized and connected via online networks of support in ways that discursively render “affective publics” (p. 2). Such affective expressions both serve to connect and distance these crowds from each other. This aspect of Twitter is an important lens through which to understand the content shared on this social networking site; it demonstrates that tweets carry not only discursive but also affective meanings, which influence people's interactions with each other and technology. Through these interactions “networked publics” emerge that are “mobilized, connected and identified, and potentially disconnected through expressions of sentiment” (p. 5). Papacharissi (2015) describes Twitter as a “medium for storytelling” (p. 2) and argues that the stories on social media serve as framing devices that allow crowds to be rendered into an affective public. In addition to Papacharissi's affective publics, we argue that story sharing on Twitter can also disrupt mainstream narratives and highlight discourses that otherwise remain invisible and marginalized in society.

While Twitter has many advantages as discussed above, one of its disadvantages is that it lacks long texts like those found in blog posts or on Facebook. Nonetheless, we would argue that even with 280 characters, Twitter users articulate strong opinions and beliefs, which form the basis of a public discussion around important social events. Even a hashtag, which is often only a few words, carries a lot of meaning and social context. Using a hashtag is contextual and therefore is a valuable point of analysis. Hashtags are meaningful “signifiers” (Papacharissi, 2015) that are woven into users' personal opinions and stories, and that get to be defined, redefined, and reappropriated. In their affective dimension, hashtags are reshared and foster connection among users.

Another important fact of Twitter is that tweets are not edit-able. This might mean that users put thoughts and intentions into their writing on Twitter, and that we can see immediate emotional responses to what is shared. The spontaneous, in-real-time responses to social events can give insights into how people react especially at the moment of a crisis. In addition, we argue that it is important to examine the tweets in relationship to their social context. Following Tufekci (2014), we see Twitter research as a useful social media space to examine because it can “provide illuminating glimpses into specific cultural and socio-political conversations” (Tufekci, 2014, p. 508).

Another perspective that can be applied to the limitations of Twitter is Herzogenrath-Amelung's (2016) argument that the “instant-response culture” (p. 1081) inherent to social media does not facilitate systematic change. While social media might bring people together, and raise awareness of certain social issues, this does not mean that systemic changes follow. Despite this notable limitation, social media can be part of other forms of resistance in modern life. While Herzogenrath-Amelung is correct that many social media activists fail to follow up on their concerns in more meaningful ways, tools such as Twitter can be critical for providing a voice to especially marginalized people, who may, for example, use it as a tool to resist mainstream discourses around racism and sexism.

### Data Collection

After the tragic event took place on October 23, 2019, we connected to the Twitter Application Programming Interface (API) to obtain the tweets pertaining to the Essex Lorry Death incident. We collected all messages that included the following hashtags #Lorrydeaths #Lorrydeath, #Essex, #Containerdeath, #Grays, #Vietnamese, #HumanTrafficking, #39deaths, #moRobinson. These hashtags were used during the 2 weeks immediately after the event took place. Specifically, we retrieved unique tweets created between October 23, 2019, and November 6, 2019, which included at least one of those hashtags. Others who may have participated in the public conversations but did not tag themselves by using the hashtags, would not have been captured by the algorithm. In total, we collected 35,158 unique tweets. Our corpus excluded retweets. Furthermore, non-unique tweets, or repeated tweets for different hashtags are counted only once. We also removed non-English tweets. In our original corpus, tweets from other languages such as Vietnamese also appeared. By manually going through a sample of those Vietnamese tweets, we recognized that they came from Vietnam based on their content. We thus removed those, and tweets in other languages such as Dutch, German, and French. Finally, we manually examined the remaining tweets to exclude irrelevant ones—for example, those that describe weather or advertise for local businesses. After applying all the filter procedures, the final dataset includes 4,376 unique tweets by 2,596 different users.

By narrowing the tweets to those in English only we focus on the discourses that this event brought up, specifically as it is relevant to discussions about Vietnamese immigrants, and undocumented immigration in the UK more generally^1^. Without the ability to limit the location of each tweet to a precise location within the UK, we argue that analyzing the English-language discourse itself provides valuable insights on attitudes toward a relatively invisible immigrant group–the Vietnamese in the UK–about which researchers otherwise have very little information. Even though we cannot know the precise location of each tweet and thus cannot confirm if each tweet was written by a UK Twitter user, we approach the article with a focus on the discourses around social issues similar to what researchers did in the analyses of Twitter reactions to Brexit debates on Twitter around secession deadlines (del Gobbo et al., 2021; Storer et al., 2021), and those of political discourses around American presidential election (Yaqub et al., 2017). This tragic event is a rare opportunity for researchers to examine the popular imagination and opinions about immigration and a relatively invisible group of immigrants in the UK.

### Structural Topic Modeling

We use structural topic modeling (STM) by Roberts et al. (2014), an automated text analysis method, to understand public expressions shared on Twitter. We conducted pre-processing standardization of the texts by treating terms within documents as bags of words, where each term represents a single feature and information on word order is discarded. Terms were also reduced to their stem form, such that “interviews” and “interviewing” become a common feature “interview.” Additionally, stop words with no semantic meaning such as “the” and “of” were removed from the corpus. Then, we applied structural topic modeling by Roberts et al. (2014) to learn about the latent topics within the corpus.

Topic models are unsupervised machine learning models, which automatically discover latent topics from text documents. A topic model presents the researcher with two crucial pieces of information: the distribution of topics within the corpus, and distribution of words per topic. In these models, a topic can be understood as a set of words representing interpretable themes, and documents are represented as a mixture of these topics. For each document, proportions across all topics sum up to 100%. For example, after fitting a model, a document might be captured by a combination of three topics: “border control” with a proportion of 70%, “human trafficking” with 20%, and other topics with 10%. We chose STM over other topic modeling methods such as Latent Dirichlet Allocation (LDA) because STM allows for variability of “word use within a topic” by a covariate (Roberts et al., 2014). In other words, in addition to representing documents as a distribution of topics, structural topic models allow the inclusion of document metadata or covariates that meaningfully affect both document-topic proportions and word distributions over topics. We incorporated time stamps of each tweet as a covariate to analyze how topic proportions vary over time.

While topic models are useful for reducing the dimensionality of text data, one challenge is that the number of topics must be chosen in advance by the researcher. As the corpus with 4,376 documents is rather small and a classification into broader themes is useful for this work, a model for 20 topics was fitted to the corpus of tweets. We performed the *searchK* function provided in the **stm** package in R (Roberts et al., 2019), which indicated that a model with 20 topics would be most appropriate because it would ensure optimal semantic coherence, the lowest residuals, and a high upper bound.^2^ We also ran multiple models of 15, 20, 25, 30, 35, and 40 topics to compare the models' exclusivity and semantic coherence metrics (Figure A2 in the Appendix), and found that a model with 20 topics ensures high semantic coherence, and relatively good exclusivity in comparison to other models. Finally, we used the package **stm insights** (Schwemmer, 2018) to visualize the topic correlations, after which we did a “deep-reading” (Nelson, 2020) of the 50 most representative tweets per topic to examine each topic in depth and created appropriate labels for each of the 20 topics.

### Computational Grounded Theory

Recent research has shown that the method STM can act as a replacement for the first level of human coding of big text data (Nelson, 2020; Rodriguez and Storer, 2020). STM fares well against hand-coding in analyzing open-ended survey responses (Roberts et al., 2014). Combining it with other qualitative methods such as narrative analysis, or discourse analysis can address its limitations such as eliminating noisy topics and provide a more nuanced analysis of the data. We approached this analysis from a computational grounded theory model and inductive approach where the themes and categories of the dataset emerge through computational pattern detection and reiterative qualitative reading (Nelson, 2020). We did this by examine the most-representative 50 tweets of each topic, which is akin to narrative analysis, to make sure that our topics make sense and are coherent. We also followed Jasso's (1988, 2004) “tripartite structure of social science analysis” and approached this article within the developing framework category to offer a valuable approach for immigration researchers to study public attitudes on social media through computational and qualitative methods.

## Results

**Figure 1:**
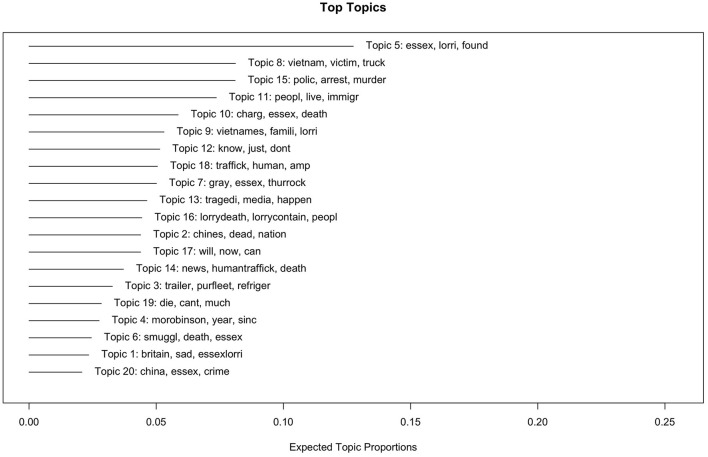
Topic proportions.

In the following section, we will provide an overview of the STM results, and then focus on the topics that are most relevant to our research question. These results offer insights into the wide range of discussions that Twitter users raised in response to the tragedy. Figure 1 shows the 20 latent topics found within the corpus. The topics are accompanied by their top three words and their proportions of occurrence in the data. The topics with the highest topic proportions include discussions of the bodies that were found (topic 5, 12.5%), questions about the victims and the fact that they are Vietnamese (topic 8, 8.1%), the murder investigation (8.1%), and illegal migration (topic 11, 7.4%). Together, they make up 36.1% of all topics.

**Figure 2:**
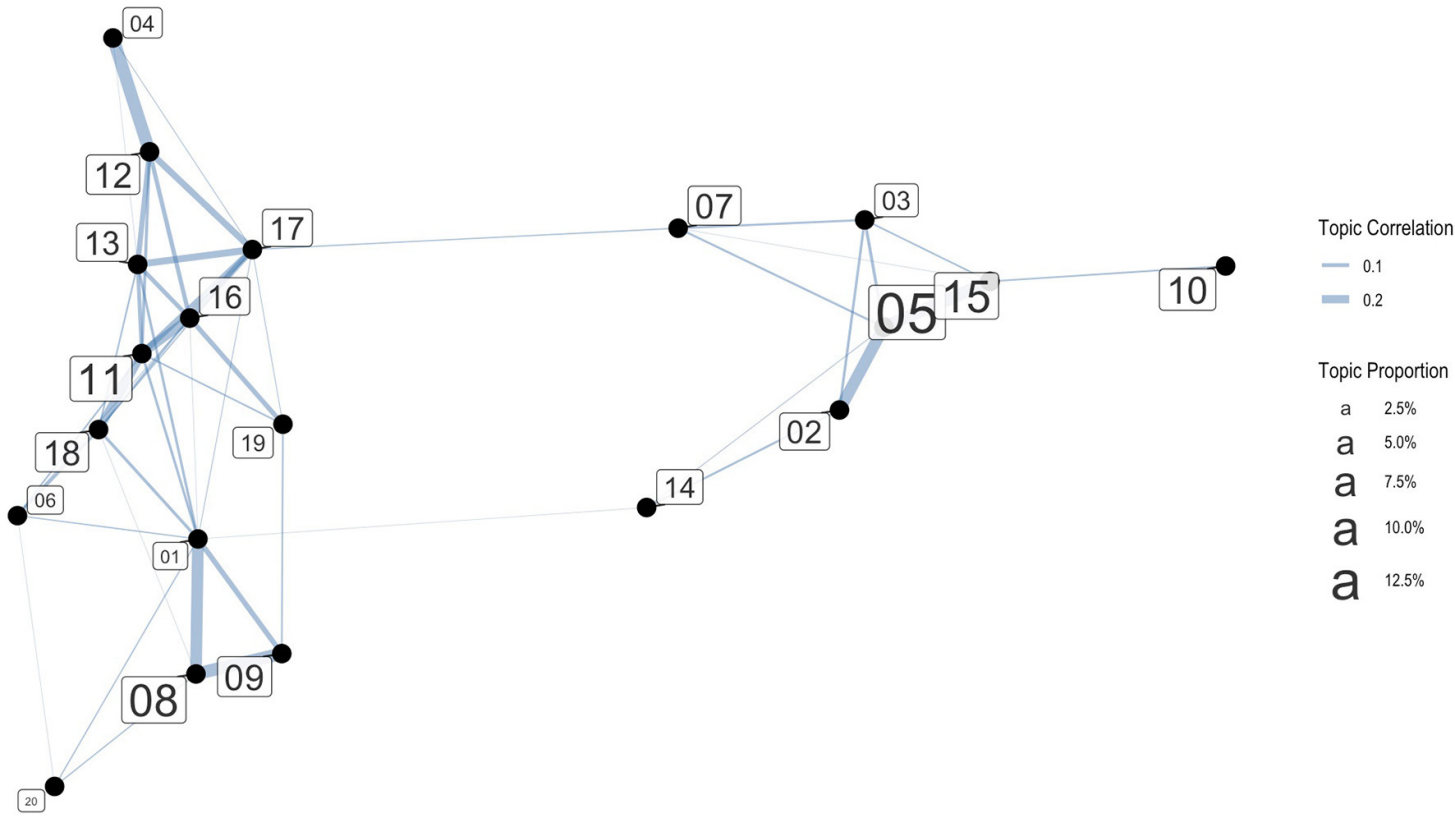
Topic correlation graph (with tie strength > 0.01). The correlated topics are indicated as connections. The connected topics are likely to occur together in one document.

Figure 2 shows the correlations between topics with tie strengths larger than 0.01. The correlated topics are indicated as connections. The connected topics are likely to occur together in one document, or in one tweet. The thicker the line between the two topics, the stronger the correlation. Figure 2 shows that there are two clusters. The smaller cluster on the right pertains to news and updates (topics 2, 3, 5, 7, 10, 14, 14), while the cluster on the left coalesces around two thematic groupings: (1) Migration narratives, stereotypes, and victim identities and (2) border control. In the qualitative analysis section, we briefly discuss the news and updates cluster before going into greater depth about the two thematic groupings mentioned above, to examine what kind of pro- and anti-immigration attitudes were expressed.

### Time Analysis

**Figure 3:**
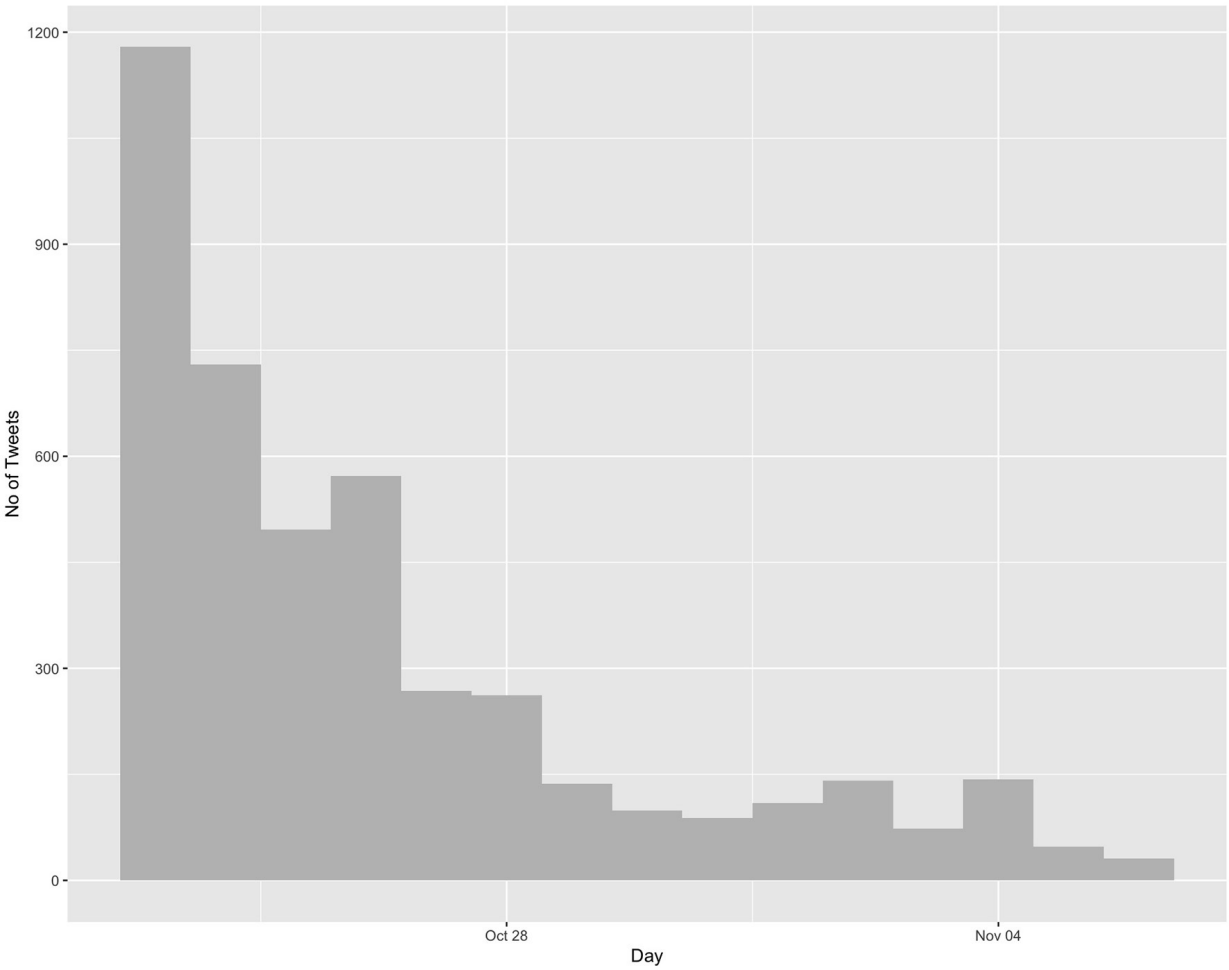
Distribution of tweets per day in the 2 weeks immediately after the Lorry Deaths incident (*N* = 4,376).

Figure 3 shows the number of tweets (*N* = 4,376) in the final dataset on the day of the tragic event and the two subsequent weeks. Most of the tweets were posted within the first 6 days of the event (total 3,507, or 80.14% of the entire dataset). In contrast, between October 29 and November 6, only 869 posts, or 19.86% of the dataset's tweets, were posted. This dwindling in the number of tweets after the first few days of the event aligns with “the overall temporal pattern of information diffusion on Twitter” (Kwak et al., 2010; Kwon et al., 2019). In the days immediately following the event, the public tweeted questions concerning the tragedy such as who was responsible for the event, who the victims were, who their families were, and where they were from. In the following days, the victims were identified as Vietnamese (October 26), and Mo Robinson, the driver, was charged with manslaughter. The victims were further identified as coming from the north central are of Vietnam, one of the poorest regions in the country. After that, the number of tweets started declining.

The number of tweets were highest during the first week after the event, and then started to dwindle in the second week. The affective public on Twitter, formed by this event, might have begun to disband because “bonds of sentiments” (Papacharissi, 2015) might fade. The affects—in particular sympathy—which the networked public shared initially, might have disappeared after the perpetrator of the event and the identity of the victims were found 2 weeks later.

**Figure 4:**
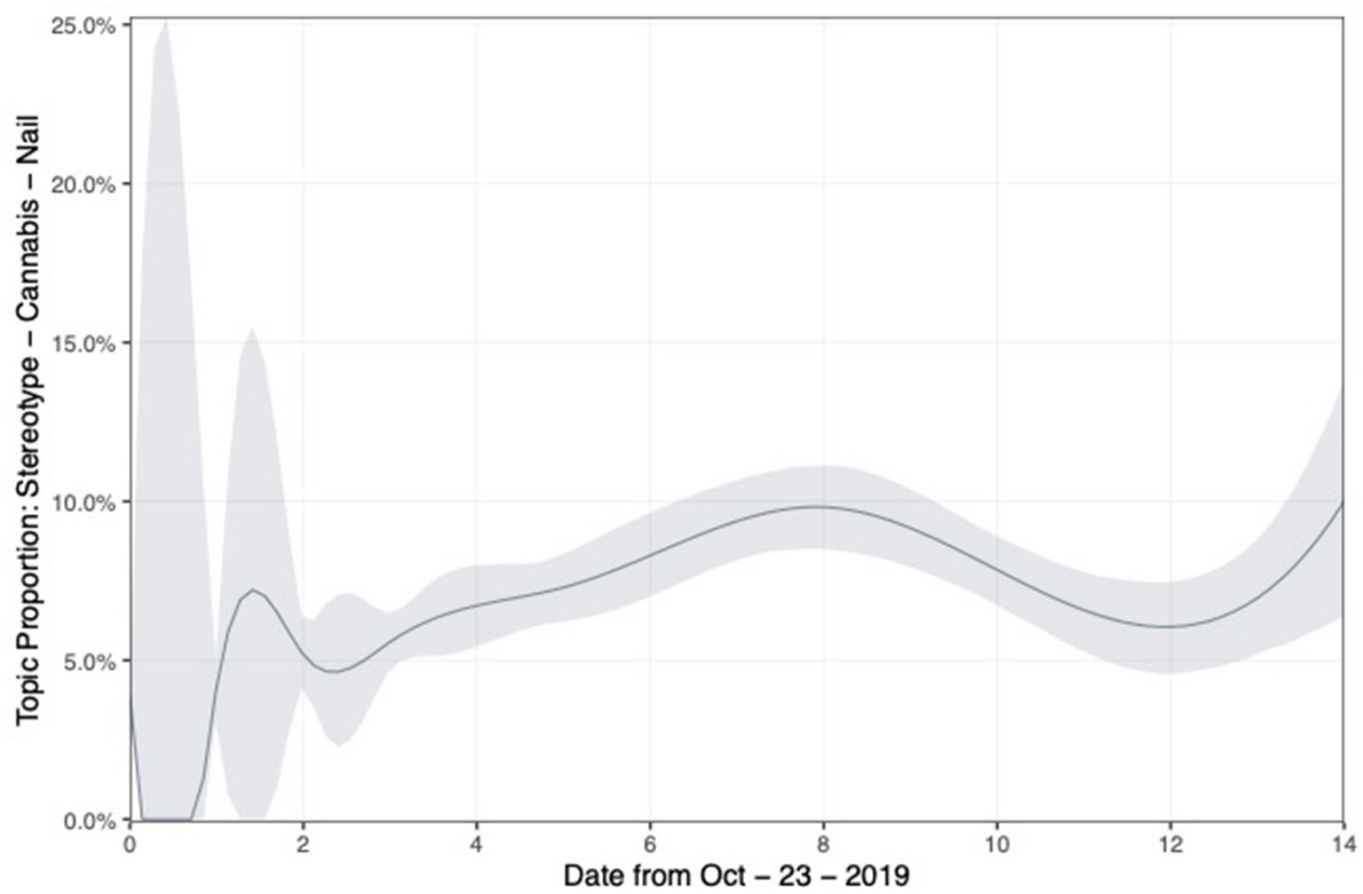
Distribution of tweets the 2 weeks after the event—Topic 18 (Vietnamese stereotypes).

While the overall number of tweets in the dataset decreased over time, a few topics' proportions increased over time. Topic 18, which concerns stereotypes of Vietnamese immigrants such as workers on cannabis farms and in nail salons, is especially interesting. Figure 4 shows the changes in topic proportion of topic 18 over time. The graph shows that this topic gained importance relative to other topics over time after day 1. The reason for the increase in discussion of Vietnamese immigrants' stereotypes relatively to other topics after 1 day of the event, is that 1 day after the tragedy the public found out that the people who had died in Essex were in fact Vietnamese, not Chinese. We analyze specific tweets concerning victims' identities and their stereotypes in the qualitative results section.

### News Updates: Disseminating Information and Sharing Sympathy

We begin the qualitative analysis with a brief discussion of the biggest group of topics, which includes topics 2, 3, 5, 7, 10, 14, and 15. This group pertains to informing the public about the tragedy, and the weight of the tragedy for British politics and international relations with the immigrant-sending country. This group of topics provides the context in which our discussion regarding Twitter users' attitudes toward immigration is situated. The topics demonstrate the different ways in which Twitter is used to spread news, and how it allows for sharing personal reactions to tragic events, such as sympathy. Prime Minister Boris Johnson, for example, wrote on Twitter:

tweet 1 [Topic 7]: I'm appalled by this tragic incident in Essex. I am receiving regular updates; the Home Office will work closely with Essex Police as we establish exactly what has happened.

This tweet by the prime minister demonstrates the severity of the event and that a politician used Twitter to keep the public informed during the investigation. He further states his feeling about it, with the term “appalled,” representing his expression of shock about the humanitarian crisis. Since the tragic event involved an investigation into many parties, countries, and locations, the spread of the news on Twitter played a vital role in the public's understanding of the Lorry Deaths incident. Twitter users were informed about the unfolding of events, including who the victims were, what the police would do with the driver, and what the victims' families would do to assuage the pain of losing their loved ones. One typical tweet in this group is the following:

tweet 2 [Topic 5]: The bodies of 39 people have been found in a lorry container in Essex. Essex Police said the lorry traveled from Bulgaria and entered the country via Holyhead, Anglesey, on Saturday.

Twitter facilitated the spread of news, and Twitter users kept the Twitter public informed about the tragedy. Twitter's algorithms suggest tweets and topics to users that are relevant at different geographical levels, including international, national, and local news. Users can follow trending topics and receive in-real time updates on the event. In “the network society” (Castells, 2011), internet users learn about crises and events increasingly through internet platforms.

Users not only received news and updates about the event, they also actively participated in showing sympathy as they shared the news:

tweet 3 [Topic 14]: These poor, desperate souls. I'm sickened by this latest case from the UK #HumanTrafficking MUST stop. #HumanRights. #HumanityFirst
tweet 4 [Topic 15]: So awful. Police say one man—believed to be the lorry driver has been arrested on suspicion of murder.

These two tweets demonstrate shared concern about the tragic event. Expressing their shock through words like “I'm sickened” or “so awful” shows Twitter users' desire to share their emotional responses to the event, and not simply to spread the news. The tweets also demonstrate an active negotiation with the tragic content and speak to research on people turning to Twitter to make sense of tragic events and expressing collective trauma (Eriksson, 2016; Fischer-Pressler et al., 2019). In addition, there is also a clear demand in tweet 3 to end human trafficking. This tweet fits within a humanitarian response that calls for “humanity first” and shows sympathy for the tragedy but does not suggest a systemic political change.

### Migration Narratives, Stereotypes, and Victim Identities

The following qualitative analysis focuses on our question about what types of public attitudes toward immigrants were evident in the Twitter discussions after the humanitarian tragedy. We focus on expressions of pro- and anti-immigrant attitudes, specifically on how pro-immigrant solidarity or sympathy expressions might implicitly reproduce anti-immigrant thoughts. This topic cluster includes topics 1, 8, 9, 13, 16, 18, and 19.

In the first few days after the tragedy, many Twitter users speculated about the identities of the victims. They raised questions about who the immigrants were, where they came from, and why they had chosen to migrate to the UK in such a dangerous way. The initial tweets reported on the identification of the victims as Chinese citizens because they carried counterfeit Chinese passports.

Critiques of the reporting by Western media on the tragedy and their spreading of false information on the identity of the victims started when it was clear that the victims were Vietnamese. Users expressed their anger with tweets like the following:

tweet 5 [Topic 13]: Many foreign media are eager to dive into Chinese society and bring to light what they believe is dark. But the danger of that is they tend to jump too fast, too soon, overlooking facts and thereby hurting their own credibility.

“Foreign media” in this case refers to Western media. This user criticized both how Western media blamed China as being partly responsible for the deaths in the lorry truck, and the media's quickness in assuming that the victims were Chinese. One of the factors that might have played into the quick conclusion by the Western media that the victims could only be Chinese could be the many tragic stories about poor Chinese migrants dying in the ocean trying to migrate to other rich countries. These stories may have foregrounded the assumption that Chinese immigrants were desperately trying to get out of China and risking their lives on the way (Chu, 2010). However, the fact that the victims carried Chinese passports was not enough to confirm their national identity. Topic 13 brings together resistance to such anti-Chinese attitudes on Twitter. Examples of this criticism include the following:

tweet 6 [Topic 13]: The Western media is really keen to turn the truck tragedy into gimmicks to attack and smear China.
tweet 7 [Topic 13]: FM [Foreign Minister] spokesperson Hua Chunying slams CNN groundlessly on #Essex truck death. Instead of focusing on the tragedy itself and how to prevent human trafficking in the future, Western media have turned this tragedy into a new stunt to defame and discredit China.
tweet 8 [Topic 13]: Illegal immigration resulted from imbalanced global development, a problem which Western media should pay more attention to rather than blaming developing countries for the tragedy happening in their own land.

These tweets emphasize the critique that Western media quickly places blame on developing countries rather than considering their own country's responsibility for the tragedy. Tweet 8 highlights how often immigration, especially immigration without documents, results from uneven economic globalization, and injustices, rather than faults of the developing countries or the immigrants themselves. This tweet is a call for solidarity with migrants and their families and a demand to dismantle the myth that migrants and migration are the crisis, and instead that “mass migration is the *outcome* of the *actual* crises of capitalism, conquest, and climate change” (Walia, 2021, p. 3). These types of critiques are counter-narratives that go beyond mainstream media reporting, which often co-opt, filter, or erase the knowledge by communities at the margin (Alemán and Alemán, 2016).

Counter-narratives or counter-story telling are practices that Critical Race Theory describes as cultivating “voices of resistance and reclamation” of communities at the margins (Alemán and Alemán, 2016, p. 289). Black Twitter is one important example of resisting and challenging mainstream media news stories about Black bodies in terms of counter-narratives (Lee, 2017) or counter-publics (Hill, 2018), often through sharing specific hashtags. While our research does not find one main hashtag that organizes resistance and the tweets of resistance are in the minority, it is important that these tweets of resistance are recognized (e.g., tweets 6–8). Such tweets demonstrate that Twitter can be a space in which users voice resistance and share counter-stories which are not reliably shared by mainstream media (Lee, 2017). Furthermore, this points to the importance of Twitter research on public opinion as tweets may offer spontaneous and real-life expressions of attitudes toward immigration that are not captured in traditional survey research.

In contrast to these counter-narratives that call for solidarity and demand to change the narrative of mass migration, other tweets blamed developing countries with authoritarian governments for emigration, without considering the colonial histories that led to these regimes. When the victims were mis-identified as Chinese, ideas circulated that those migrants had had to flee an authoritarian regime. This idea was also advanced when it became known that the migrants had migrated from Vietnam:

tweet 9 [Topic 1]: Authoritarian regimes as the root cause of these tragedies should also be stopped and extinguished. R.I.P. to the 39 victims #Vothitramy #VietNam. Essex truck deaths: Parents of Vietnamese woman feared dead say they were told it was a 'safe route' - CNN

This Twitter user calls for the extinction (“extinguishing”) of authoritarian regimes and blames such regimes as the “root cause” for such tragedies. Here again, the blame for the Lorry deaths is put on a developing country instead of contextualizing such tragedy as part of a global migration system tied to colonialism and border control. The Twitter user cites CNN and Twitter as a way to provide others with news updates. Twitter users are therefore responding to the news media discourse and sharing their thoughts on it. One Twitter users critiqued the anti-China idea that the country has an authoritarian regime and challenged the narrative of Chinese backwardness:

tweet 10 [Topic 13]: One wonders what's going on in UK/US regarding the Anti-China narrative? US, UK companies need China. UK Mainstream Media still try to make out China is a backward authoritarian regime as if Mao was still alive. That was the narrative on #lorrydeaths! Why?

When the victims were identified as coming from Vietnam, a poorer country than China, and specifically from the northern central area of Vietnam, one of the poorest regions in the country, the narrative about migration motives changed. The narrative switched from running away from a repressive regime, to running toward economic opportunities. Many attributed the victim's reason for migration to poverty.

tweet 11 [Topic 1]: Vietnam is a booming economy but not everyone is benefitting from it. It has a huge surplus of labor. While the country has achieved reductions in #poverty, it is unequal across groups & regions. #HumanTrafficking #Vietnamese
tweet 12 [Topic 11]: Migratory pressures are encouraging millions to make long and dangerous journeys to escape suffering and poverty and to seek safer and better lives in countries like #Britain #Essex #Belgium #NorthernIreland #Holyhead

Others discussed the labor conditions that Vietnamese migrants would find in the UK, which were based on exploitative practices ranging from underpaid jobs in “nail bars” to being “exploited as slaves” in “cannabis factories”:

tweet 13 [Topic 18]: Cannabis factories in the UK sustained by human trafficking children; teenagers kidnapped from Vietnam; exploited as slaves.
tweet 14: [Topic 18]: Vietnamese victims of trafficking may end up working in nail bars, so what's the solution? We told it's time for labor inspection agencies to have proper funding so it can do its job in protecting workers from abuse.
tweet 15 [Topic 18]: Vietnamese victims of human trafficking may end up being underpaid and overworked in nail bars. Many would say we need an ethical stamp or similar – that's not the right solution.

In these tweets, Twitter users critique the exploitative practices of the human trafficking cycle between the UK and Vietnam. Here, users charge several actors for sustaining this cycle: the human traffickers, UK cannabis factories, and UK labor inspection agencies. A related discussion concerned human trafficking as a cause for the deaths and exploitation of immigrants:

tweet 16 [Topic 18]: The former UK Antislavery Commissioner has criticized the UK approach to tackling Human Trafficking and Modern slavery, stating the need for greater investigative resources and a stronger focus on tackling the Organized Crime networks involved.
tweet 17 [Topic 18]: The deaths of 39 migrants found in a truck in #Essex, UK has put the spotlight on trafficking, slavery in the nail bar sector, a common destination for trafficked Vietnamese.

These discussions suggest a search for those responsible for the Essex Lorry Deaths among the human traffickers and the failures of the UK government. One of the demands is to increase interventions into “the organized crime networks involved” (tweet 16). This demonstrates a desire for increased criminalization of human trafficking and organized crime. It demonstrates some trust that the UK government would address these crimes. However, these critiques do not address more structural issues such as UK's immigration policies and can therefore still be read as anti-immigration; these tweets do not actually suggest more humane and welcoming immigration policies, which would reduce the risks involved in immigrating in irregular ways.

Furthermore, although these tweets critique exploitative labor practices and the violence of human trafficking, they also reify two stereotypes about Vietnamese immigrants. First, they rely on the stereotype that Vietnamese immigrants in the UK immigrate via human trafficking. In fact, there are four groups of Vietnamese migrants to the UK: “refugees from the former South Vietnam, refugees from the former North Vietnam, new undocumented economic migrants from northern and central Vietnam, and international students” (Barber, 2020, p. 10). Second, they reproduce a stereotype that all Vietnamese immigrants work in exploitative spaces like nail bars and cannabis factories. The reality here, too, is more complex. Many nail salons are owned by Vietnamese entrepreneurs, many of whom are first-generation immigrants and from northern Vietnam. However, this concentrated entrepreneurship in nail salons is due to the support of family and friends already in the industry, as well as “structural disadvantages,” including a lack of access to other employment sectors and “an avoidance of racism” in other sectors (Barber, 2020, p. 9). Therefore, even though these tweets challenge some exploitative labor practices, they offer an inadequate explanation about what is broken in the UK immigration system. Thus, these seemingly pro-immigrant tweets can reproduce harmful stereotypes and anti-immigrant ideas.

Twitter users also blamed the victims' families for the incident:

tweet 18 [Topic 11]: Are none of the networks asking WHY these families sent their sons/daughters here for £30,000 illegally, when for a fraction of that they could have visited here, met with colleges, got advice on visas, looked at student/work options, made useful contacts? #Purfleet
tweet 19 [Topic 19]: Why can't these people, if their families have access to that kind of money, come here on a visit, do some research, make contacts, ask about work placements or higher education, apply for a visa? #Purfleet
tweet 20 [Topic 19]: If people are texting their families from inside a container & have paid smugglers 30,000 & the families are aware what is happening then who is to blame?

Each of these Twitter users saw the families as responsible for their family members' deaths. They justified such statements by highlighting the large sum of money (30,000 British pounds) that human traffickers demanded for smuggling people to Europe. The tweets demonstrate a lack of understanding of the reasons why people migrate and the mechanisms of international migration. The immigrants who died in the lorry truck were driven to escape the poorest region in Vietnam. On a structural level, this is consistent with uneven development and globalization where core economies like the UK have the most capital and developing countries like Vietnam supply resources and excess labor leading to economic migration from developing countries to the UK (Massey et al., 1993). Immigration scholars have found that economic decisions for migrating are familial decisions, in which the family pools resources together to send one member abroad (Massey et al., 1993). The blaming of families sending their loved ones to Europe fails to acknowledge the structural and historic factors that produce immigration globally, as well as the fact that the hardening of borders increases the likelihood of injuries and death for people crossing them.

In contrast to the anti-immigrant attitudes expressed through the blaming of families, other Twitter users expressed their sympathy and condolences to the victims and their families. Along with sharing condolences, one Twitter user expressed their dissatisfaction with the border system, while another expressed their sadness and sympathy about the deaths and imagined the despair they must have felt to go on such a journey:

tweet 21: [Topic 16]: Essex. Obviously, they were trying to flee to the UK but got frozen to death at the back of the lorry. Shame how humankind created borders and make it hard for its own kind. My respect and condolences to those who have lost their life.
tweet 22: [Topic 16] The scale of tragedy is too hard to understand or comprehend. My heart breaks for the families of these poor souls. No one knows why they were crammed into the steel lorry container, but one can guess how desperate they must have been.

Another Twitter user shared their heartbreak and demanded that responsibility for the tragedy be assumed and decided upon:

tweet 23 [Topic 16]: Heartbreaking to read the last words of Pham Thi Tra to her mum. A despicable end to a hope for a better life. Breaks my heart. Somebody must be made to pay. God Bless young lady.

And yet another user pointed to the continuity of racism and hate toward immigrants:

tweet 24 [Topic 16]: They can die in the back of a lorry, drown in the sea but it won't change some who will still hate them and celebrate their desperate deaths.

### Border Control

In this section, we dive into the cluster of topics that coalesce around the idea of responsibility. The cluster include topics 3, 4, 6, 11, 12, 17, 20. Many Twitter users questioned who was responsible for such a horrendous tragedy. Is it the immigration system, the tightening of the borders, or human traffickers who are to blame?

After the tragic event took place, Twitter users critiqued immigration policies and the lack of safe immigration options. Many tweets were critical of migration regimes and the criminalization of migration, and one even compared it to the criminalization of abortion:

tweet 25 [Topic 17]: This is what can happen when the government shut down safe & legal routes of entry and when they militarize borders. 'We now need more than warm words from ministers - people need to be able to access the UK safely.' - on the horrific situation in #Grays, Essex.

The tweet presents a critique of militarization of borders and calls on politicians to provide concrete actions, rather than simply saying “warm words.” This tweet emphasizes that expressions of sympathy are not sufficient in the context when people died because of stringent and punitive border policies. Instead, substantial changes are needed to prevent future tragedies.

tweet 26 [topic 11]: People who are forced to take dangerous and sometimes fatal passages to reach the UK often do so because current immigration policies and practices deny them safe and legal options.

This Twitter user points out that the UK's existing immigration system does not allow for safe immigration, particularly for some groups of people, i.e., low-skilled immigrants coming from countries outside of the European Union. This ties back to Burnett's (2017) discussion on discourses in the UK that otherize migrants, particularly Eastern European migrants as well as racialized others. The system is predicated on the idea that some migrants are more deserving than others, and those who are not deserving are excluded from the system. Such exclusion leads to dangerous attempts to migrate through illegal routes such as what the victims in this tragedy had attempted.

tweet 27 [Topic 11]: The UK needs to face up to the global refugee crisis and create safe, legal routes for refugees and migrants – tightening border controls simply creates a market for human trafficking gangs and people risk more dangerous journeys to better their lives.

Similar to the tweets above, this tweet demands that the UK, as a rich and powerful nation, should assist with “the global refugee crisis” by creating “legal and safe” channels for immigration. This tweet demands responsibility from the UK immigration system to help refugees and migrants in the long term.

tweet 28 [Topic 11]: The horror in Essex yesterday shows that criminalizing migration is like criminalizing abortion: it doesn't make it stop, it only makes it criminal. And like abortion, desperate people will always try and find a way to evade the law, often with terrible results.

This Twitter user compares the act of criminalizing immigration to criminalizing abortion. Underlying this argument is that both immigration and abortion are fundamental human rights, but that the state can punish and criminalize certain activities. This criminalization does not stop migration and instead makes it more unsafe.

Overall, these tweets highlight the position that without a safe migration route and legal ways for immigrants to come and reside in the UK, people resort to dangerous options like human trafficking, which are both harmful to migrants themselves and create issues for law enforcement. These Twitter users emphasize how the lack of migratory pathways inevitably created the Lorry deaths tragedy. Without changing the existing infrastructure, more tragedies would be unavoidable.

Such tweets demonstrate that border control is central to the debate about who is responsible for the tragic deaths. Andersson (2014) argues that the border policy in the European Union has given rise to an industry that promotes illegality outside of the EU borders. One example for this militarization of the EU borders and the criminalization of migration is Frontex and its evolving security practices (Léonard and Kaunert, 2020). Building on that argument in the US context, Massey et al. (2016) found that the militarization of borders in the United States exacerbated undocumented immigration. When the US government intensified military control of its borders, immigrants would find other ways to get into the US, and most of the time these ways would be more dangerous, such as hiring coyotes to traffic them into the country. Furthermore, circular migration would stop because going home became much more dangerous. Therefore, several Twitter users critique both the increasing militarization of the UK border and Brexit, and the resulting increase in unsafe routes of migration. They voice their opinions regarding immigration policies through rigorous discussions and political analyses. Thus, Twitter is an important source of public opinion because it contains more nuanced debates by Twitter users, which cannot be captured by survey data.

Yet, other Twitter users took the opposite position, blaming the tragedy on the laxity of border patrols. They advocated for a tougher border control regime to manage and reduce the amount of illegal border crossings, thus decreasing the number of undocumented immigrants in the UK:

tweet 29 [Topic 3]: Recently in an airport, my luggage passed twice through a scanner, monitoring everything I was possibly bringing inside and on board. What is it then that makes it impossible to scan for human beings on board of trucks before crossing the Channel? #39people #Zeebrugge #Purfleet

This tweeter thinks that an increase of surveillance at checkpoints other than airports would prevent such a tragedy.

tweet 30 [Topic 4]: Prevention is better than cure! Remove the incentives for illegal immigrants! Welfare reform & immediate removal of illegals would dramatically reduce the numbers trying to enter illegally!! #lorrydeaths

The Twitter user evokes the anti-immigrant trope that immigration is a burden to the UK welfare system (Alfano et al., 2016). Therefore, this tweet supports the position that to prevent such tragedy, the UK needs to have tougher immigration policies including increasing deportation.

Still others debated whether Mo Robinson, the driver, and his associates should be blamed:

tweet 31 [topic 4]: I wonder how many people he has smuggled in before, where are they now? Are they being used for prostitution? Being abused? How much money has he made? Defend him and know your defending a criminal. #MoRobinson

Tweet 31 questions how many people beyond the 39 victims in this case were transported by Mo Robinson in the past. He financially benefited from the suffering of these victims. His involvement in transnational human trafficking should not be tolerated.

tweet 32 [Topic 12]: Hearing Mo Robinson has been released without charge. Hopefully this is the case. Poor lad and family's name tarnished forever. Calling someone a mass murderer and making him notorious worldwide is a disgrace when none of the facts where available. He called 999! Justice for Mo.

This tweet frames Robinson as the victim and argues that he is innocent till proven. The Twitter user calls for “Justice for Mo.” This is a kind of co-opting of the language of social movements. Twitter users' expressions of solidarity with the Essex Lorry death victims used the #justiceFORthe39” to demand justice for those who died (for example in a tweet in Topic 13)^3^.

tweet 33 [Topic 12]: Honestly, I hope the media are held to account if the Lorry Driver in the Lorry deaths case is found to be innocent. Naming and showing photo of suspect so early on pre-court hearing is disgraceful and unfair. Should he be found innocent I hope he takes them to the cleaners.

This tweet similarly defends the driver, arguing that he is innocent until proven guilty. Furthermore, the Twitter user criticizes the role of the media in spreading photos of the suspect so early on, thus leading to his trial in the court of public opinion before the real court hearing.

Other Twitter users accused organized Chinese criminals of smuggling the people who died:

tweet 34 [Topic 6]: Organized Chinese criminals called snakehead gangs are well known for people smuggling, prompting some to speculate that they may have a hand in the deaths of 39 people found in a refrigerated lorry trailer in Essex.
tweet 35 [Topic 20]: Snakehead travel agents in China are transporting people to Europe for 6-26k to work in black market syndicates, according to organized crime expert Dan Silverstone.

These two tweets try to justify their accusations by citing an expert (as in tweet 35). The accusations also evoke anti-Chinese stereotypes and may be related to the initial suspicion by the Western media that the victims were Chinese, as discussed above.

The incident also stirred up more general xenophobia among a smaller but still vocal group of tweeters. Concerns ranged from fear of crime to competition for jobs and social services, sometimes called “welfare chauvinism” (Eger and Breznau, 2017), typified in the following tweets:

tweet 36 [topic 11]: I have zero sympathy toward 39 illegal aliens infiltrating our borders and potentially at risk of putting more native folks on the streets homeless. Draining our systems. #lorrycontainer #gmb #realist.
tweet 37 [topic 11]: Prevention is better than cure! Remove the incentives for illegal immigrants! Welfare reform: immediate removal of illegals would dramatically reduce the numbers trying to enter illegally!

These two examples use the same anti-immigrant tropes that were put forth by the Brexit campaign—in particular, the idea that immigrants take away employment and cause poverty to “native folks” (tweet 40). Other anti-immigrant attitude was shared regarding resources:

tweet 38 [topic 11]: #Lorrydeaths if you can afford 30 grand to come here, why not stay put where you are. There aren't many people here who could put that sort of money together.
tweet 39 [Topic 11]: Not likely many genuine refugees, asylum seekers pay $50,000 for people smuggling and people trafficking. Is Vietnam really bad? How many safe countries passed through to get to Brexit oops UK?

These Twitter users argue that immigrants could not be poor because they could afford “30 grand” to come to the UK. Tweet 39 claims that “genuine refugees” would not be able to afford such an expensive passage. There are two tropes implied in this argument: the undeserving immigrant, and victim blaming. The language around authenticity constructs migrants as those forced to migrate while others do so voluntarily (or unnecessarily). In line with Flores Morales and Farago (2021) study on (un)deserving undocumented immigrants, this tweet evokes ideas of the deserving and undeserving immigrant. Here, the deserving one is constructed as one who is poor and cannot in any way get a large sum of money to pay human traffickers. In addition, the tweet claims that the immigrants themselves are to be blamed for their own deaths. Victim-blaming literature speaks to this phenomenon where the media or legal spaces blame ethnic and racial minority groups for lack of equal access to resources or poverty. Moody-Ramirez and Cole (2018), for example, found that some Twitter users participated in victim blaming by using traditional stereotypes of Black men and portraying the violence against them as isolated cases rather than as part of broader systemic injustice.

## Discussion

This study's contributions are three-fold: (1) it demonstrates the utility of Twitter for public opinion research, as well as the value of using computational social science to study social media; (2) it offers a more nuanced understanding of how pro-and anti-immigration attitudes are expressed in response to a tragic event; and (3) it expands the limited literature on public attitudes toward Vietnamese immigration to the UK.

One of the contributions of our research is the power available from using a computational approach. Using automated text analysis and qualitative “deep reading,” we engaged in a “holistic interpretation” (Nelson, 2020) of tweets within the context of the post-Brexit immigration debate. We investigated Twitter conversations in the wake of the Lorry Deaths tragedy, an incident in which 39 undocumented Vietnamese migrants died inside a lorry container in the UK. Using computational tools, we collected Twitter users' discussions related to the Essex tragedy (*N* = 4,376). Structural topic modeling discovered 20 latent topics, which we then used in a “deep reading” of selected tweets (Nelson, 2020) analyzing each of them in relation to the post-Brexit British immigration debates.

Through the visualization of the topic correlations of the entire corpus, we found two main clusters, which we labeled “news and updates,” and immigration debates. The immigration debate cluster was further thematically grouped into two more nuanced groupings: (1) migration narratives, stereotypes, and victim identities, and (2) border control. Our analysis of the news and updates cluster suggests that Twitter users amplified the spread of news as events unfolded. Alongside sharing the news, some users added their concerns and sympathies regarding the incident.

This paper shows the strengths of Twitter as a platform for public opinion research. As Twitter users responded to an event in real-time, their tweets expressed immediate reactions to the tragedy, which are often implicitly and explicitly tied to immigration debates more broadly. Tweets encapsulated diverse opinions and attitudes toward complex issues such as immigration policies in only 280 characters. We show that some Twitter users, even when expressing sympathies toward victims, reproduce racist and xenophobic tropes through stereotyping and anti-immigration narratives, such as the belief that immigrants abuse the UK's social welfare system.

Our research highlights the importance of a nuanced understanding of how pro- and anti-immigration attitudes manifest on social media in response to a tragedy involving immigrants. The qualitative analysis of tweets shows that Twitter users expressed both anti- and pro-immigration attitudes. Discussions shifted along with new information that emerged from further investigation. A major debate in the first few days was about the Western media's misidentification of the victims' national identities. Here, tweets critiqued the Western media as too quick to blame China for the tragedy. The discussion then shifted to expressions of sympathy to victims because they came from an impoverished region in Vietnam. Twitter users also engaged in counter-narratives that challenged the mainstream media's reporting of the event. We highlight these counter-narratives because they show “resistance and reclamation” of communities at the margins (Alemán and Alemán, 2016, p. 289). Tweeters challenged the mainstream narrative that developing countries were responsible for the migration crisis. Rather, the true causes were structural factors such as inequality, and uneven global development, conditions that developed countries are actively creating and exacerbating in a global scale.

Furthermore, Twitter users discussed the dangers of the human trafficking business. In these posts, we found repeated references to two stereotypes about Vietnamese immigrants: the idea that all Vietnamese immigrants come to the UK through human trafficking, and that all Vietnamese work in nail salons or on cannabis farms. We argue that these tweets reify anti-Vietnamese tropes in more subtle, perhaps inadvertent ways. Other anti-immigration tweets blamed families for the deaths of their family members. This put the blame on individual people rather than acknowledging the historical factors that have contributed to global migration flows.

In our analysis of Twitter discussions on border control, we also found expressions of both anti- and pro-immigrant attitudes. On the one hand, there were critical perspectives on immigration pathways, border control, and the connection between stricter border regimes and violence. Twitter users pointed out that the existing immigration system was exclusionary, and that it did not provide options for some groups of potential migrants, such as low-skilled immigrants coming from outside of Europe. They argued that this structural condition forced potential immigrants like the victims of the Lorry Deaths incident to resort to dangerous means of human trafficking. Users also criticized a punitive border regime where crossing borders is criminalized. They demanded that politicians must act to resolve such important problems instead of simply expressing sympathy to victims in public. On the other hand, we found defense of the truck driver's innocence, a call for more surveillance at border checkpoints of cargo and blame of the victims for their own deaths.

Our results on anti-immigrant attitudes also pertain to research on the media representation of immigrants. McKay et al. (2011), for example, found that media reports on the arrivals of asylum seekers on boats in Australia depicted them as pursuing economic gain and exploiting Australians; humanitarian concerns around the reasons why asylum seekers had migrated were largely absent from these accounts. There are both consistencies and inconsistencies in our results concerning McKay et al.'s (2011) findings. Even though some Twitter users portrayed the immigrants who died in the Lorry Deaths incident as threats to the UK, many also expressed sympathy and critiques of strict border regimes. There are two possible explanations for the differences in findings. First, due to the tragic deaths, sympathy and humanitarian concerns might have been heightened compared to coverage of events in which undocumented immigrants are alive when they arrive. Second, McKay et al. (2011) analyzed public responses to media reports. The demographic of people using Twitter and those responding to media reports might be different, since it is not just journalists writing on Twitter but everyday people, politicians, and other actors who use Twitter to publicly share their opinions.

In another content analysis of newspaper coverage of a tragic immigration event, in which 359 people died in Lampedusa, Zerback et al. (2020) found that the initial tone of coverage of immigration issues changed. After Lampedusa, there was an increase in immigration coverage, immigrant actors, and the number of stories depicting immigrants as victims in the news. There was also an increase in positive depictions of immigrants as well as their journeys to Europe. But these changes only lasted for a short time. Similarly, we found that the intensity of the coverage on Twitter of the Essex Lorry Deaths incident decreased significantly within a short period. This raises the question of whether such tragic events influence immigration attitudes beyond the subsequent days or weeks. Our analysis revealed that both anti-and pro-immigrant voices were galvanized by the tragedy.

Czymara and Schmidt-Catran (2017) find that, against their expectations, no changes occurred in the acceptance of immigrants, specifically Muslims, after the New Year's Eve sexual assaults in Cologne, Germany in 2015/2016. Even though anti-Muslim racist tropes circulated in the aftermath of the event, there was little to no evidence of increases in anti-immigrant attitudes in the survey data analyzed. This might suggest that events that might seem able to shift the public discourse are not as strongly affecting medium- or long-term public opinion. The existence of both anti-and pro-immigrant attitudes within our data likely reflects the polarized context of the UK, in which a virulent debate about Brexit was ongoing. Future research should address the issue of whether meaningful shifts in pro- or anti-immigration attitudes occur in the longer term after tragic or impactful events.

Finally, the discourses about Vietnamese immigrants that we analyze in our research add to the growing literature about ethnic minorities in the context of increasing diversity in the UK (Vertovec, 2007), such as studies by Barber (2015) and Song (2021), regarding public opinion about an often-forgotten immigrant group in the UK, the Vietnamese.

### Limitations

There are limitations to our approach. First, using hashtag queries might exclude tweets which are a part of the conversation, even though they did not use any hashtags. Second, we excluded tweets in other languages. The victims, their families, and communities speak another language (Vietnamese). The Vietnamese community in the UK could have tweeted in Vietnamese instead of English, and by defaulting to English, we potentially excluded their points of view. Excluding tweets in Vietnamese may have produced unknown sampling bias. Finally, although Twitter is a useful source for examining public conversations, there is evidence that data collected via the Twitter API could be biased. The API only allows access to 1% of the total tweets posted in real time. The reason for the selection of this 1% threshold is unknown to researchers outside of Twitter. This filter could create additional unknown biases.

Furthermore, the relatively small number of tweets in our dataset might point to a finding itself: the lack of coverage of Vietnamese immigrants to the UK. Both the rather small amount of Twitter coverage and the fact that Twitter responses decreased relatively quickly within 2 weeks further points to the public's lack of interest in Vietnamese immigrants to the UK and their continued invisibility (Barber, 2020).

Despite limitations in data collection, and modeling, our study offers rich interpretations of large-scale conversations on Twitter. Our analysis offers a unique contribution to the literature on attitudes about immigration by using social media to study public opinion about immigration issues and by providing in-depth analysis of how an unsupervised machine learning method could uncover complex discourses around immigration and border issues.

## Data Availability Statement

The code for reproducing our results can be found at https://github.com/ngathan/essex_lorry. Data is available upon request. Other inquiries can be directed to the corresponding author.

## Author Contributions

NT: study conception and design and data collection. NT and FW: analysis and interpretation of results. NT, FW, and LS: draft manuscript preparation. All authors reviewed the results and approved the final version of the manuscript.

## Conflict of Interest

The authors declare that the research was conducted in the absence of any commercial or financial relationships that could be construed as a potential conflict of interest.

## Publisher's Note

All claims expressed in this article are solely those of the authors and do not necessarily represent those of their affiliated organizations, or those of the publisher, the editors and the reviewers. Any product that may be evaluated in this article, or claim that may be made by its manufacturer, is not guaranteed or endorsed by the publisher.

## Footnotes

^1^We did not set a geographical filter on Twitter because only ~1–3% of tweets contained geolocation information (Graham et al., 2014; Osborne and Dredze, 2014). This translates to only 43–131 tweets in our dataset having precise locational information. To recover geographical locations, researchers use methods ranging from using users' self-identified locations, reference locations, Gaussian mixture models, to language modeling to recover the locations of tweets. However, there is no one single solution to recover all tweets' locations in a precise manner.

^2^See Appendix A1 for further explanation about how the number of topics (K) was chosen.

^3^Topic 13: A massive thank you to all the amazing folk who turned out to pay their respects to their fellow humans who died in pursuit of a better life.” #essex39 #justiceforthe39 #folkestone #weareallhuma.
