# Supplementary material for: #Lorrydeaths: Structural Topic Modeling of Twitter Users' Attitudes About the Deaths of 39 Vietnamese Migrants to the United Kingdom
Source: Front Sociol. 2022 Aug 11;7:787450. doi: 10.3389/fsoc.2022.787450 (PMC9404524; doi:10.3389/fsoc.2022.787450)
Supplement: Supplementary file 1 [file Data_Sheet_1.docx]

# Appendix (Online Supplement) for “**#Lorrydeaths: Structural Topic Modeling of Twitter Users’ Attitudes about the Deaths of 39 Vietnamese Migrants to the United Kingdom**”

**Figure A1:** Results of the SearchK function in STM


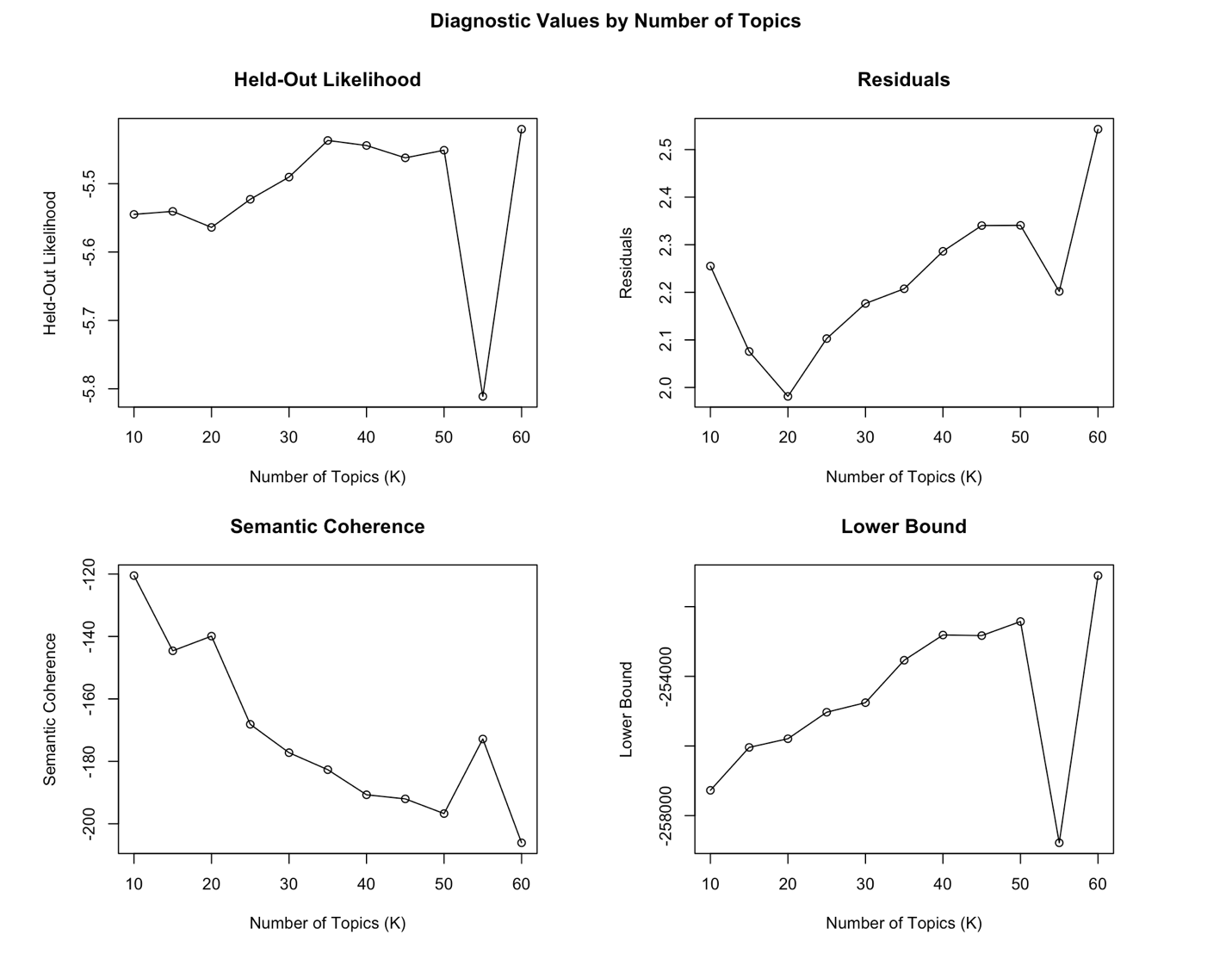


Figure A1 shows how different models of various K number of topics fit in relation to each other. “Held-out” refers to the process of partitioning the original data into two sets: a training set, and a test set. The STM algorithm creates the topics and their distributions over the corpus using the train set. Then the test set, without any topics assigned to each document, is used to test the accuracy and recall of the training set results including topic distributions and word distributions of each topic. The held-out likelihood is the log probability of topics in the test set correctly replicating topics of the training set. Ideally, this number should be high. The “lower bound” refers to the lower limit of the marginal log likelihood. “Residual” refers to the difference between expected and predicted topic predictions. Semantic coherence measures the co-occurrence of words in each topic. Co-occurring words also comprise the most likely words of a given topic. When a topic has high semantic coherence, the words used to define the topic have a high likelihood of reflecting the same topic. This figure shows that K = 20 produces low residuals, high semantic coherence, and high lower bound, though held-out likelihood is not much different from K in the interval between 10 to 25. For each model, we also added the timestamp when each tweet was posted online as a covariate.

**Figure A2:** Model Diagnostics.


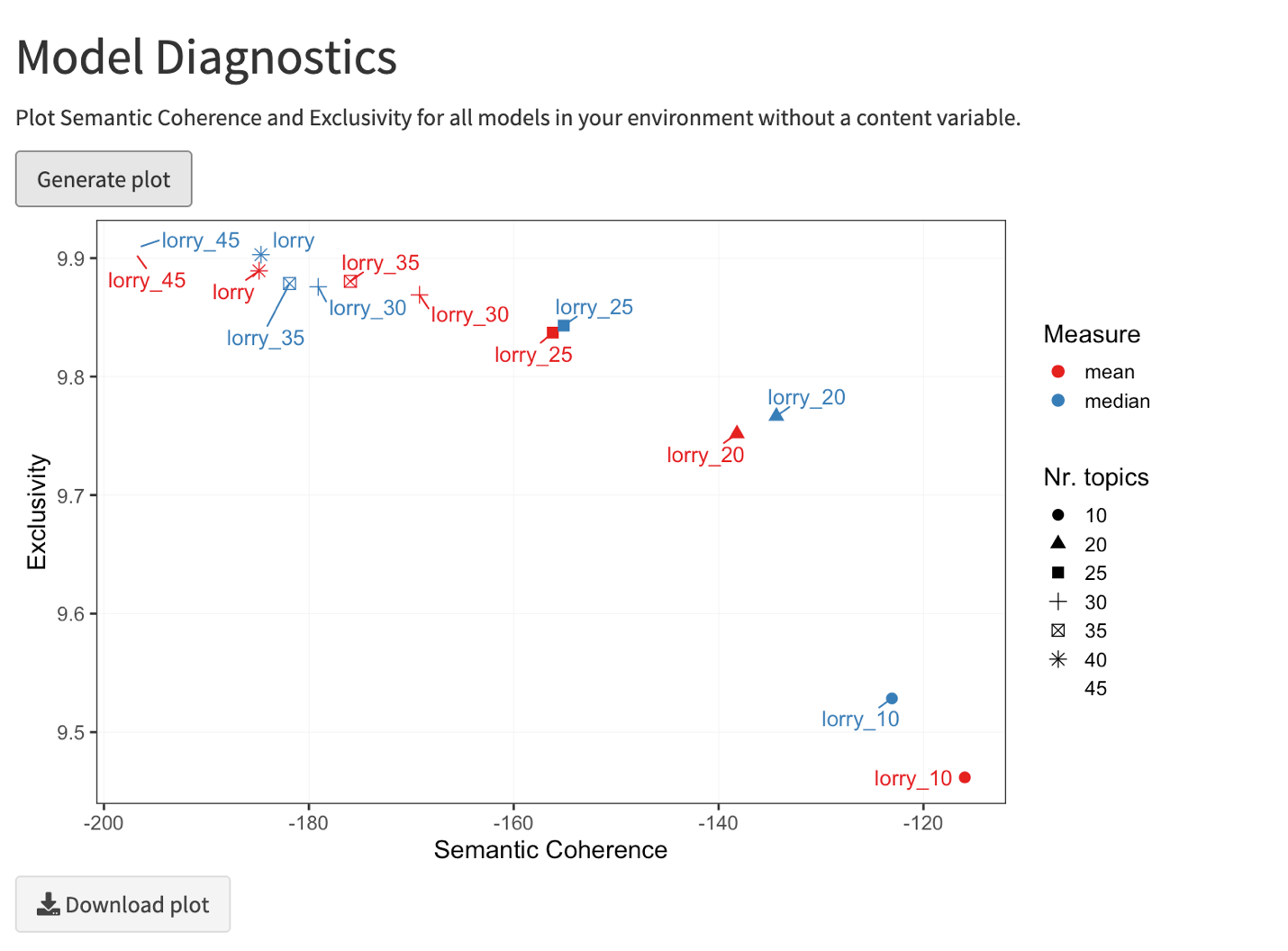


Figure A2 maps semantic coherence against exclusivity. Exclusivity refers to the probability for a word to fall primarily within the top rankings of a single topic. A model where neither metric dominates is a good model. In this case, K = 20 is the appropriate choice.

**Table 1:** Table of 20 topics with their proportion. The most frequent terms used for each topic determined by the FREX metric (Lucas et al. 2015).

| **Topic** | **Themes** | **Label** | **Percentage** | **Example** |
| --- | --- | --- | --- | --- |
| 2 | News Updates | Chinese_nationals | 4.4 | Essex lorry deaths: 39 found dead were Chinese nationals #England #Essex #Lorry #Deaths #Bodies #Chinese |
| 3 |  | Migration_route | 3.3 | The police have confirmed the trailer arrived in the docks in #Purfleet without a unit, Mo picked the trailer up out the docks where these ILLEGAL IMMIGRANTS were already in the trailer!! He had the trailer for only 35 minutes #JusticeForMo!!  Is Mo Robinson innocent? The trailer is registered in Bulgaria, to a company owned by a lady from Northern Ireland. |
| 5 |  | bodies_found | 12.7 | 39 dead bodies found inside lorry in #Essex |
| 7 |  | Tragedy_discovered | 5 | Horrific news coming out of Essex today. Thoughts with all the families of those involved and the emergency services who have had to deal with what must have been a very distressing scene. |
| 10 |  | manslaughter_charge | 5.9 | Driver of lorry charged with 39 counts of manslaughter. Mo Robinson from Craigavon will appear in court on Monday. Also charged with conspiracy to traffic people & money laundering. #Essex |
| 14 |  | Breaking_news | 3.7 | "The discovery is among the worst of its kind in the UK since 2000, when the bodies of 58 Chinese people were found in a container at Dover in Kent." #HumanTrafficking #Chinesenationals #ukpolitics #illegalimmigration |
| 15 |  | Murder investigation | 8.1 | A murder investigation launched after 39 people were found dead in a truck container at Waterglade Industrial Park in Grays, #Essex. A 25-year-old man from Northern Ireland has been arrested on suspicion of murder; police confirmed. |
| 8 | Migration Narratives, Stereotypes & Victim Identities | Vietnamese | 8.1 | 39 UK truck victims likely from #Vietnam The majority of the 39 people found dead in the back of a truck near #London were likely from Vietnam, a community leader from the rural, rice-growing community where many of the victims are believed to |
| 1 |  | Vietnam action | 2.3 | Dread builds in #Vietnam over fate of missing UK #amigrants. Le Minh Tuan last heard from his 30-year-old son Le Van Ha in a Facebook message that read: "I'm about to board a car to Britain. I will contact the family when I arrive in #England, Dad." Read> |
| 9 |  | Victim families | 5.3 | Latest on #Essex lorry deaths: - 30 Vietnamese families report missing relatives - All of the families are from north-central provinces - Nghe An (18), Ha Tinh (10), Quang Binh (1) and Thua Thien-Hue (1) |
| 13 |  | China | 4.6 | Many foreign #media are eager to dive into #Chinese society and bring to light what they believe is dark. But the danger of that is they tend to jump too fast, too soon, overlooking facts and thereby hurting their own credibility, says. #lorrydeaths |
| 16 |  | Sympathy to victims | 4.4 | #Essex Obviously they were trying to flee to the UK but got frozen to death at the back of the lorry. Shame how humankind created borders and made it hard for its own kind. My respect and condolences to those who have lost their life |
| 19 |  | Victims last words | 2.8 | So sorry mum and dad. The route to abroad didn't succeed. Mum. I love you and dad so much. I am dying because I can't breathe. I am from Can Loc Ha Tinh. Vietnam. Mum. I'm very sorry. #lorrydeaths |
| 18 |  | Cannabis nail trafficking | 5 | Trafficked #Vietnamese and the lure of nail bars and cannabis farms - learn about the push and pull factors of #THB from #Vietnam. Good to hear the voice of as experts in this field in the #ecpat |
| 11 | Border Control | Illegal migration | 7.4 | This is what can happen when the govt shut down safe & legal routes of entry and when they militarise borders. 'We now need more than warm words from ministers - people need to be able to access the UK safely.' - on the horrific situation in #Grays, Essex |
| 4 |  | Mo Robinson guilty | 2.8 | Mo Robinsons GF's Salon, Dottie Avenue IG, should also be investigated for money laundering using proceeds from crime. From Council to Luxury in 3 years. Jillcoulter1989 knew where that money was coming from 100% #Morobinson #DottieAvenue #justiceFORthe39 |
| 12 |  | Mo Robinson innocent | 5.1 | So I didn't agree with the man's name and other personal details being released so early on as if he wasn't charged his life would have been ruined! His details at first were the only details they had right at first! But now has been Charged it looks like jail time! #morobinson |
| 6 |  | Smuggler’s cruelty | 2.5 | Organised Chinese criminals called snakehead gangs are well known for people smuggling, prompting some to speculate that they may have a hand in the deaths of 39 people found in a refrigerated lorry trailer in #Essex. |
| 17 |  | Responsible parties | 4.4 | Responsibility lies first and foremost with the criminals who organised the transport of these individuals, more details of which we will probably learn in the coming days and weeks #Essex |
| 20 |  | Crime Transport | 2.1 | I'm horrified by the death of 39 human beings in #Essex in a #humantrafficking crime. Those who are uber #nationalist about #Refugees, #Borders, #immigrants, etc. are guilty of creating the circumstances that allow this crime against #humanity. People are legal. #Aliens is #BS |
